# Evaluation of the adverse event profile and pharmacodynamics of toceranib phosphate administered to dogs with solid tumors at doses below the maximum tolerated dose

**DOI:** 10.1186/1746-6148-9-190

**Published:** 2013-09-30

**Authors:** Luis Feo Bernabe, Roberta Portela, Sandra Nguyen, William C Kisseberth, Michael Pennell, Mark F Yancey, Cheryl A London

**Affiliations:** 1 Department of Veterinary Biosciences, College of Veterinary Medicine, The Ohio State University, Columbus, OH 43210, USA; 2 Department of Clinical Medicine, College of Veterinary Medicine, University of Illinois, Champaign-Urbana, IL, Champaign; 3 Department of Veterinary Clinical Sciences, College of Veterinary Medicine, The Ohio State University, Columbus, OH 43210, USA; 4 Division of Biostatistics, College of Public Health, The Ohio State University, Columbus, OH 43210, USA; 5 Zoetis, Kalamazoo, MI 49007, USA

**Keywords:** Toceranib phosphate, VEGF, Dog, Cancer

## Abstract

**Background:**

The receptor kinase inhibitor toceranib phosphate (Palladia) was approved for use in dogs in 2009 using a dose of 3.25 mg/kg administered every other day. Preliminary data suggests that lower doses of toeceranib may be associated with a reduced adverse event profile while maintaining sufficient drug exposure to provide biologic activity. The purpose of this study was to determine the Cmax of toceranib in dogs with solid tumors receiving 2.5-2.75 mg/kg every other day and to document the adverse events associated with this dose rate. Secondary objectives included determination of plasma VEGF concentrations in treated dogs and response to therapy.

**Results:**

Dogs with solid tumors were administered toceranib at an intended target dose ranging from 2.5-2.75 mg/kg every other day and plasma samples were obtained for analysis of toceranib and VEGF plasma concentrations on days 0, 7, 14 and 30 of the study at 6 and 8 hours post drug administration. Additionally, plasma samples were obtained at 0, 1, 2, 6, 8, and 12 hours from dogs on day 30 for confirmation of Cmax. Response to therapy was assessed using standard RECIST criteria and adverse events were characterized using the VCOG-CTCAE. Toceranib administered at doses between 2.4-2.9 mg/kg every other day resulted in an average 6–8 hr plasma concentration ranging from 100–120 ng/ml, well above the 40 ng/ml concentration associated with target inhibition. Plasma VEGF concentrations increased significantly over the 30 day treatment period indicating that VEGFR2 inhibition was likely achieved in the majority of dogs. The lower doses of toceranib used in this study were associated with a substantially reduced adverse event profile compared to the established label dose of 3.25 mg/kg EOD.

**Conclusions:**

Doses of toceranib ranging from 2.4-2.9 mg/kg every other day provide drug exposure considered sufficient for target inhibition while resulting in an adverse event profile substantially reduced from that associated with the label dose of toceranib. This lower dose range of toceranib should be considered for future use in dogs with cancer.

## Background

Toceranib phosphate (Palladia) is the first FDA approved receptor tyrosine kinase inhibitor for the treatment of cancer in dogs [1]. Toceranib acts by blocking the function of several receptors on the surface of tumor cells, as well as on the surface of new blood vessels including vascular endothelial growth factor receptor 2 (VEGFR2), platelet derived growth factor receptor (PDGFR), and stem cell factor receptor (KIT), permitting it to have effects on both the tumor as well as the tumor microenvironment (vasculature, etc.) [1-5]. While toceranib was approved based on its demonstrated activity against canine mast cell tumors (MCT), activity against various other tumor types has been documented including several types of carcinomas [1,3,6].

The label dose for toceranib (3.25 mg/kg given every other day, EOD) was determined in the initial Phase I clinical trial that established the maximum tolerated dose (MTD) [3]. Adverse events (AEs) associated with this regimen included loss of appetite, lethargy, diarrhea, vomiting, weight loss, neutropenia, and lameness [1,3]. Furthermore, during the pivotal clinical field study of toceranib in dogs with MCT using the 3.25 mg/kg EOD dosing regimen, approximately 20% of dogs had a dose reduction and 50% needed a drug holiday due to AEs [1].

Based on work performed in laboratory dogs as well as pharmacokinetic sampling from dogs in the Phase I study [3,7,8], 3.25 mg/kg of toceranib given EOD provides sustained plasma drug concentrations at or above 40 ng/ml, the presumed necessary threshold for receptor inhibition *in vivo* as established in rodent studies [4]. Furthermore, in a pharmacodynamic study performed in dogs with MCT, a dose of 3.25 mg/kg of toceranib was found to produce plasma concentrations of drug at 8 hours post administration ranging from 30–180 ng/ml [5]. In this study, intratumoral target modulation as evidenced by downregulation of KIT and/or extracellular signal-regulated kinase (ERK) phosphorylation was demonstrated at toceranib plasma concentrations as low as 30 ng/ml at 8 hours post drug administration.

Clinical evidence exists that biologic activity occurs when doses below the MTD of toceranib are used in dogs with a variety of tumors. For example, in the Phase I study, of 16 dogs treated with toceranib at 2.5 mg/kg EOD, 6 (37.5%) had an objective response to therapy (4 complete responses, CR and 2 partial responses, PR) with an additional 5 dogs experiencing stable disease (SD) [3]. This compared favorably to 20 dogs treated at 3.25 mg/kg EOD in which 8 had an objective response to therapy (2 CR, 6 PR) with an additional 4 dogs experiencing SD. These data provided preliminary evidence that lower doses of toceranib may provide sufficient drug exposure to elicit anti-tumor activity, which could then result in fewer AEs permitting more continuous drug administration.

Following its approval, toceranib was used off label in a variety of tumor types. A retrospective study of this use found evidence of biologic activity in anal gland anal sac adenocarcinomas (AGASACA), thyroid carcinomas, head and neck carcinomas, and nasal tumors [6]. The objective response rates in these tumor types varied from 25-75%, with the clinical benefit rate (CR + PR + SD) exceeding 80%. The median dose of toceranib used in these dogs was 2.8 mg/kg and over half the dogs were treated on a Monday/Wednesday/Friday (MWF) dosing regimen instead of EOD [6]. Interestingly, while the incidence of diarrhea was comparable to that reported in the clinical field study of toceranib in MCTs [1], the rates of anorexia, vomiting, lethargy, and neutropenia were substantially lower. Together, these data provide evidence that lower doses of toceranib may be sufficient for target inhibition while reducing the AE profile.

Although pharmacokinetic analyses have been performed in both healthy dogs and dogs with cancer as part of the Phase I study and in a small number dogs in the MCT pivotal field study [3,7], there has been no coordinated study of toceranib plasma concentrations when intentionally given at lower than the label dose. Therefore, the purpose of this study was to assess the presumed maximum concentration of drug (Cmax) in dogs with cancer receiving an intended dose of 2.5-2.75 mg/kg of toceranib EOD and to evaluate changes in plasma VEGF concentrations during the course of treatment.

## Methods

### Eligibility

This clinical trial was approved by the Clinical Research and Advising Committee at the College of Veterinary Medicine and Institutional Animal Care and Use Committee at Ohio State University. Dogs with a sarcoma, carcinoma, melanoma or other solid tumor that had either failed standard therapy or for which the owner declined standard therapy were eligible for enrollment into this study. Tumor types excluded from this study were MCT, hemangiosarcoma, histiocytic sarcoma and lymphoma. Additional eligibility criteria included age of at least 1 year, an estimated life expectancy of at least 90 days, adequate organ function as indicated by standard laboratory tests, at least 2 weeks since prior investigational therapy, chemotherapy, radiation therapy, or surgery, and no evidence of any serious systemic disorder (e.g., cardiac disease) considered incompatible with the study.

### Drug product and concomitant medication

Toceranib phosphate was provided by Pfizer Animal Health (now Zoetis) in 10 mg, 15 mg, and 50 mg size tablets. Concomitant medications considered acceptable for use to prevent and/or treat drug related AEs included famotidine, omeprazole, metronidazole, loperamide, metoclopramide, ondansetron, maropitant, tramadol, carprofen, meloxicam and prednisone.

### Study design

A total of 40 dogs were enrolled in this study, with dogs initially assigned to toceranib at 2.5 mg/kg or 2.75 mg/kg EOD. All dogs were placed on famotidine at 0.5-1 mg/kg once per day at study initiation and owners were instructed to administer the toceranib in the morning with food. Physical examination, routine bloodwork (CBC, biochemistry profile) and blood sampling for assessment of toceranib and VEGF plasma concentrations was performed pretreatment and on days 0, 7, 14 and 30 of treatment. On days 0, 7, and 14, blood was collected at 6 and 8 hours post drug administration to coincide with the predicted toceranib Cmax based on previous studies that had established the time of maximum drug concentration (Tmax) to be 5.3-9.3 hours [7,8]. On day 30, blood sampling was performed at 0, 1, 2, 6, 8, and 12 hours post drug administration to confirm either 6 or 8 hours as the true Cmax. Owners of dogs that experienced CR, PR, or SD at study day 30 were offered the option of continuing toceranib administration for an additional 6 months (provided at no cost), although patient data was not officially captured beyond day 30.

### Assessment of adverse events

Dogs were evaluated for AEs on days 0, 7, 14, and 30 of study, as well as at interim time points as needed. AEs were defined and graded according to the published VCOG-CTCAE criteria [9]. Disease progression or signs and symptoms definitely related to disease were not considered AEs.

### Tumor response assessment

Tumor assessments were performed on days 0, 7, 14 and 30 of study. Tumor assessment was performed using caliper measurement with digital photography documentation, thoracic radiography, ultrasonography, or spiral computerized tomography (CT) as indicated. Determination of antitumor efficacy was based on objective tumor assessments made according to the RECIST v.1.1 guidelines using unidimensional measurements [10]. Response to therapy was defined as complete response (CR, resolution of all target and non-target lesions, no new lesions), partial response (PR, at least 30% decrease in the longest diameter of target lesions, no progression of non-target lesions, and no new lesions), stable disease (SD, decrease in target lesions of less than 30% or increase of target lesions less than 20%, no progression of non-target lesions, and no new lesions) or progressive disease (PD, greater than 20% increase in target lesions, progression of non-target, new lesions). Dogs were defined as experiencing clinical benefit if they had CR, PR, or SD.

### Blood collection and processing

Blood was collected into EDTA tubes and centrifuged at 3000 × g for 10 minutes. Plasma was then collected and split evenly into two portions in cryovials, then stored at -80°C until analysis was performed for toceranib and VEGF concentrations.

### Analysis of toceranib plasma concentrations

Toceranib was quantitated in plasma samples using the method previously described [7,8]. Briefly, 50 μL aliquots of plasma were fortified with an internal standard (IS), a deuterated toceranib analog. Toceranib and the IS were extracted from the plasma by protein precipitation using acetonitrile. The analyte and IS are light sensitive and undergo photoisomerization when exposed to light. Therefore, the 96-well plates were protected from light by wrapping the bottom and sides with foil and securing with adhesive tape. Calibration standards (1 to 1000 ng/mL) and quality control (QC) samples were prepared with separate weighing of toceranib reference material (Pfizer, Kalamazoo, MI, 98.1% purity). Standard and QC solutions were prepared by serially diluting a stock solution (100 μg/mL in methanol) into control canine plasma so that no standard or QC sample contained more that 1% methanol. Study samples were analyzed as single replicates.

The prepared samples were injected onto a Zorbax StableBond CN column and elution of toceranib and the IS accomplished with an acetonitrile:15 mM ammonium formate solution (pH 3.25) 30/70 v/v mobile phase with a 80/20 v/v rinse for 2.3 minutes at 5 minutes after each injection. Detection was accomplished using MS/MS using a Sciex 4000 triple-quadrupole mass spectrometer with Turboionspray ionization source (MDS Sciex, Toronto, Canada). Transition ions of m/z 397.2 → 283.2 for toceranib and 401.3 → 283.2 for the IS were used. The standard curves were generated using peak area ratios of toceranib to the IS and a 1/concentration^2^ weighted quadratic regression. Toceranib concentrations were determined by interpolation over the quantitation range of 1.00 ng/mL to 1000 ng/mL.

### VEGF plasma concentrations

A solid phase sandwich ELISA was used to measure plasma concentrations of VEGF. The manufacturer’s protocol for Canine VEGF Quantikine ELISA kit (R&D Systems) was followed using 200 μl of undiluted plasma and 100 μl of standard. The standard was serially diluted to create a range of 4.88 pg/ml to 1250 pg/ml. All standards and samples were performed in duplicate and the absorbance reading at 540 nM was subtracted from the absorbance reading at 450 nM.

### Statistical analysis

VEGF concentrations were analyzed in terms of percent change from baseline. Repeated measures ANOVA (with the Greenhouse-Geisser correction to account for lack of compound-symmetry) was used to determine if percent change in VEGF differed between 0, 7, 14, and 30 days. Percent change in VEGF (dependent variable) was also related to Cmax and Tmax, (independent variables) using separate linear mixed models containing animal-specific intercepts and slopes and a power correlation structure for the residual errors to allow within animal correlations to differ with proximity of measurements. The Bayesian Information Criterion (BIC) was used to select between linear, quadratic, and cubic trends in our mixed models. Repeated Measures ANOVA was performed using Intercooled Stata Version 11 (Stata Corp, College Station, TX) and the linear mixed models were fit using PROC MIXED in SAS Version 9.2 (SAS Inc, Cary, NC).

## Results

### Demographics

A total of 40 dogs were enrolled in the study between August 2010 and December 2011. All dogs were treated and followed at The Ohio State University Veterinary Medical Center. Thirty-nine dogs completed the study; one patient did not finish the study as the owner elected euthanasia. Baseline characteristics for the 40 dogs are presented in Table 1. Median age upon study enrollment was 9.8 years and the median body weight was 30.5 kg. The majority of dogs entered were mixed breed dogs. Tumor types included soft tissue sarcoma (n = 7), AGASACA (n = 7) thyroid carcinoma (n = 7), nasal carcinoma (n = 5), squamous cell carcinoma (n = 4), primary pulmonary carcinoma (n = 3)*,* metastatic osteosarcoma (n = 3), biliary carcinoma (n = 1), poorly differentiated carcinoma in the neck region (n = 1), salivary gland adenocarcinoma (n = 1), and mediastinal carcinoma (n = 1). Fifteen dogs had received prior therapy consisting of surgery, chemotherapy, and/or radiation therapy, and 25 dogs had not received any prior treatment.

**Table 1:** Patient demographics

**Patient demographics**	
**Age (yr)**	
Median	9.8
Range	5-13.5
**Weight (kg)**	
Median	30.5
Range	14.3-62.7
**Gender (n)**	
Male intact	3
Castrated male	23
Female spayed	14
**Tumor type (n)**	
Soft tissue sarcoma	7
Thyroid carcinoma	7
Anal sac adenocarcinoma	7
Nasal carcinoma	5
Squamous cell carcinoma	4
OSA (lung metastasis)	3
Primary pulmonary carcinoma	3
Billiary carcinoma	1
Poorly differentiated carcinoma (neck and jaw)	1
Salivary gland adenocarcinoma	1
Mediastinal carcinoma	1
**Prior therapy (n)**	
Yes	15
No	25

### Dosing of toceranib

Dogs were initially assigned a dose of either 2.5 mg/kg or 2.75 mg/kg. However, given tablet size limitations, the dose rates were not exact, and this was further compounded by changes in body weight during the study as well as the need for an occasional dose adjustment due to AEs. Table 2 shows the actual mean doses of toceranib for dogs enrolled in the study determined by averaging the dose calculated over the four study visits. These are grouped by range of dose, and the number of dogs in each dose range is provided. The number of dogs requiring a dose modification was five for those initiated at 2.5 mg/kg and three for those initiated at 2.75 mg/kg.

**Table 2:** Toceranib dosing groups

**Dose range (mg/kg)**					
**2.35-2.39**	**2.40-2.49**	**2.50-2.59**	**2.60-2.69**	**2.70-2.79**	**2.80-2.89**
2.35	2.40	2.50	2.62	2.70	2.89
2.38	2.41	2.50	2.62	2.70	
2.39	2.47	2.50	2.63	2.72	
	2.47	2.51	2.63	2.72	
		2.51	2.66	2.73	
		2.51	2.68	2.75	
		2.53	2.68	2.78	
		2.54	2.68	2.78	
		2.55	2.69		
		2.56			
		2.57			
		2.57			
		2.58			
		2.59			
		2.59			

### Response to therapy

Tumors were assessed at baseline and again on days 7, 14 and 30 or at the time of suspected progression by direct measurement, thoracic radiography, abdominal ultrasound or CT. SD over 30 days was observed in 31/40 dogs (77.5%) and 5/40 dogs experienced PR to therapy (12.5%) for an overall clinical benefit rate of 90%. There were 4 dogs (10%) that developed PD during the course of treatment. These data are summarized in Table 3.

**Table 3:** Response to therapy by tumor type

**Tumor type**	**N**	**PD**	**SD**	**PR**
Soft tissue sarcoma	7	2	5	
AGASACA	7		7	
Thyroid carcinoma	7		5	2
Nasal carcinoma	5	2	3	
Squamous cell carcinoma	4		2	2
Metastatic osteosarcoma (lung)	3		3	
Primary pulmonary carcinoma	3		3	
Billiary carcinoma	1		1	
Poorly differentiated carcinoma	1		1	
Salivary gland adenocarcinoma	1			1
Mediastinal carcinoma	1		1	
**Total**	40	4	31	

*PD* Progressive Disease *SD*, Stable disease *PR* Partial response.

### Adverse events

Drug-related AEs were limited and in most cases readily treatable with supportive care. Using the VCOG-CTCAE classification [9] there were 188 grade 1, 27 grade 2, 8 grade 3, and 1 grade 4 AEs (Table 4); these are described below:

**Table 4:** Adverse events in toceranib treated dogs

**Adverse event**	**Grade 1**	**Grade 2**	**Grade 3**	**Grade 4**
Diarrhea	34	6		
Vomiting	23	3		
Neutropenia	20	1		
Lethargy	15	2		
Anorexia	15	1		
Anemia	13			
Increase ALP	9	2	1	
Increase BUN	9	1		
Increase ALT	7		1	1
Lameness	6	8	1	
Increase AST	5		1	
Protein losing nephropathy	4			
Cough	4			
Epistaxis	3			
Panting	3			
Ocular discharge	3			
Weight loss	3			
Hypertension	1		2	
Thrombocytopenia	2			
Hypopigmentation	2			
Increase CK	2			
Colitis		2		
Hand-foot syndrome	1			
Dermatitis	1			
Alopecia	1			
Ileus			1	
Pancreatitis			1	
Fever		1		
Pruritus	1			
Constipation	1			
Constipation	188	27	8	1

#### *Gastrointestinal*

Fifteen dogs (52.5%) developed grade 1 diarrhea during the study, 10 of which experienced more than one episode. Six dogs developed grade 2 diarrhea, and two had grade 2 colitis. Eighteen dogs (37.5%) developed grade 1 vomiting, 5 of which experienced more than one event; only 3 dogs experienced grade 2 vomiting. Thirteen dogs (32.5%) developed grade 1 anorexia and 1 had grade 2 anorexia. Lethargy was encountered in 13 dogs (32.5%), 11 of which were grade 1 episodes with the remainder grade 2 in severity. Only 3 dogs had weight loss, ranging from 5-10% of their initial recorded body weight, consistent with a grade 1 event.

#### *Hematologic*

In 42.5% (20/40) of the dogs, grade 1 neutropenia was documented after 1–3 weeks of toceranib therapy; only 1 episode of grade 2 neutropenia was documented in all dogs. These events were not associated with infection or fever and in most instances the neutropenia resolved over time. In those cases where neutropenia persisted during the course of therapy, the grade did not worsen. Thirteen dogs experienced grade 1 anemia during the study, 6 of which had a mild anemia of chronic disease prior to beginning toceranib administration. Only 2 dogs developed grade 1 thrombocytopenia.

#### *Biochemistry abnormalities*

Increased blood urea nitrogen (BUN) without a concomitant increase in creatinine was noted in 9 dogs, of which 6 had concurrent gastrointestinal upset. Transient grade 1 elevations in alanine aminotransferase (ALT) were noted in 7 dogs and not associated with any specific clinical signs; these resolved spontaneously without discontinuation of therapy. Five dogs had mild increases in aspartate aminotransferase (AST, grade 1) and 1 dog developed a grade 3 AST elevation at the end of the study period. Alkaline phosphatase elevations (grade 1, n = 10, grade 3, n = 1) were observed as well. Of note, no dogs enrolled in this study developed clinical evidence of hepatotoxicity.

#### *Neuromuscular*

Mild- to-moderate lameness (grade 1–2) occurred in 10 dogs; 1 dog experienced grade 3 lameness, (severe with occasional weight bearing on the affected limb). Physical examination in affected dogs often revealed focal muscle pain, but no obvious joint or bone pain. The lameness resolved in all cases with rest, pain medication (e.g., tramadol, carprofen, meloxicam) or a change in the toceranib dose.

#### *Other adverse events*

Other side effects experienced by dogs enrolled in this study included ocular discharge, coughing, epistaxis, panting, hypertension, pancreatitis, ileus, protein losing nephropathy, and various skin disorders. For the majority of these attribution was unlikely to be associated with toceranib; rather, the events were either a consequence of disease and/or due to other causes (i.e., concomitant medications, etc.). Grade 3 hypertension developed in 2 dogs; discontinuation of toceranib and initiation of enalapril resolved the event in one dog, while the other dog’s hypertension was believed secondary to pain associated with the tumor and the development of pancreatitis. In this dog, pancreatitis was diagnosed based on clinical signs (acute vomiting, lethargy, weakness, and an elevated cPLI at 470 ug/L). Although the dog was improving clinically with supportive care, the owners elected euthanasia due to a poor prognosis associated with the tumor (soft tissue sarcoma attached to the thoracic wall in the axillary region). A full necropsy was performed and although gross findings appeared consistent with pancreatitis, this was not confirmed upon histopathologic examination of the pancreas.

There were 4 patients that developed protein losing nephropathy (PLN) without an apparent cause, one of which had concurrent hypertension. All patients showed an inactive sediment and absence of blood in the urine. Three out of these 4 patients continued on toceranib after the study and went on to receive enalapril +/- amlodipine; the proteinuria remained stable despite continued therapy.

One dog on study developed depigmentation and thinning of the footpads in all 4 limbs. This was associated with pain/hypersensitivity when walking. The toceranib was discontinued for 1 week at which time the AE resolved and drug was continued at a lower dose.

### Management of adverse events

The adverse events discussed above were primarily grade 1 and 2 in nature and in nearly all dogs resolved with the addition of concomitant medications and/or temporary discontinuation of toceranib administration. Medications used to treat/prevent these toxicities included famotidine, omeprazole, metoclopramide, maropitant, ondansetron, metronidazole, loperamide, enalapril, tramadol, prednisone and NSAIDs (carprofen or meloxicam). In addition to the dog that presented with pancreatitis, one other dog required hospitalization due to vomiting and diarrhea of one-day duration. This resolved with fluids, metoclopramide, famotidine, metronidazole, and ondansetron and toceranib administration was re-initiated without further complication.

### Analysis of toceranib plasma concentrations

A primary objective of this study was to evaluate the presumed Cmax of toceranib given at doses below that indicated on the label (3.25 mg/kg) to determine whether these doses were associated with concentrations of drug likely to result in biologic activity. In prior studies the Cmax was determined to be approximately 6–8 hours post drug administration [7,8]. Additionally, a pharmacodynamic study in dogs demonstrated that plasma concentrations of toceranib 8 hours post drug administration ranging from 30 to 180 ng/ml were associated with target modulation *in vivo*[5].

**Figure 1:**
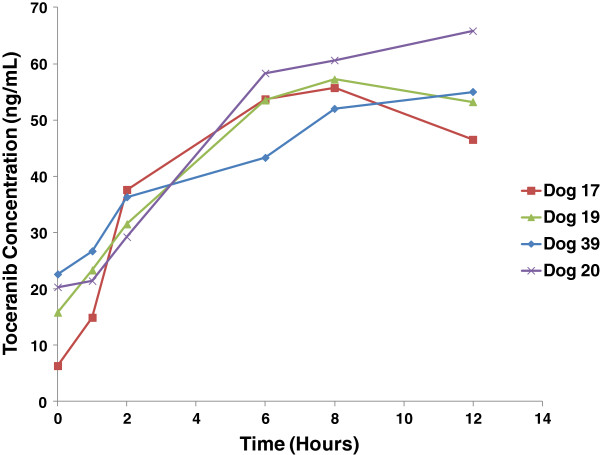
**Toceranib plasma concentrations on day 30 of treatment.** Shown are representative plasma toceranib concentrations from 4 dogs obtained on day 30 over 12 hours following drug administration. In this study, 28/40 dogs had Cmax at 6 or 8 hours on day 30. Two of the dogs shown experienced Cmax at 8 hours while the other two experienced Cmax after this time.

**Figure 2:**
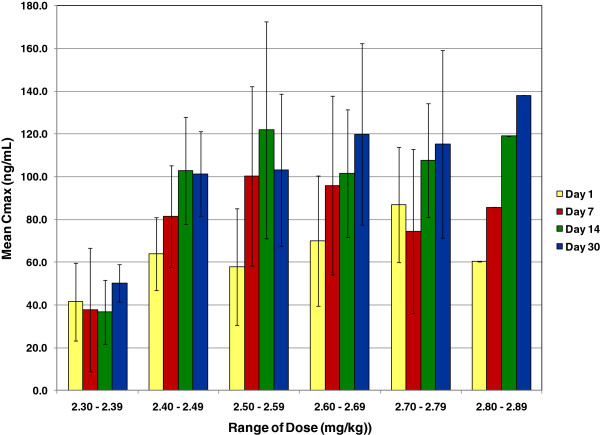
**Toceranib Cmax concentrations.** Shown are the median Cmax concentrations of dogs treated with toceranib in this study. These are presented for each time point of collection (days 0, 7, 14, and 30) by dose range. For all dose ranges above 2.4 mg/kg, Cmax was above the 40 ng/ml concentration of toceranib predicted to be associated with effective target inhibition.

The plasma concentrations of drug at 6 and 8 hours were measured and the highest value was used as the presumed Cmax at days 0, 7 and 14. The plasma samples taken at day 30 over 12 hours confirmed that this was true for 69% (28) of the dogs who had a Cmax at 6 or 8 hours. Of the 12 dogs with Cmax at 12 hours, the reported Cmax ranged from 51% to 98% of the 12 hour concentration: 5 were at > 90%, 8 were > 80%, 10 were > 75% of the 12 hour concentration. Figure 1 shows representative plasma toceranib concentrations at day 30 from 4 dogs. Therefore, for all dogs the plasma concentration reported as Cmax was the true maximum plasma concentration of toceranib post administration, or a value < 25% lower than the Cmax. These values are shown in Figure 2, grouped by dose range. The presumed Cmax plasma concentration of toceranib increased gradually in most dogs over the first 2 weeks of dosing despite the EOD regimen and appeared to level out by day 30. Toceranib dose ranges of 2.4-2.9 mg/kg were associated with mean presumed Cmax values of 100–200 ng/ml by day 30, well above the 8 hour 40 ng/ml plasma concentration considered to be sufficient for target inhibition *in vivo*. Lastly, there did not appear to be significant differences in drug exposure in dogs dosed between 2.4-2.9 ng/ml by day 30 of dosing.

### Plasma VEGF

**Figure 3:**
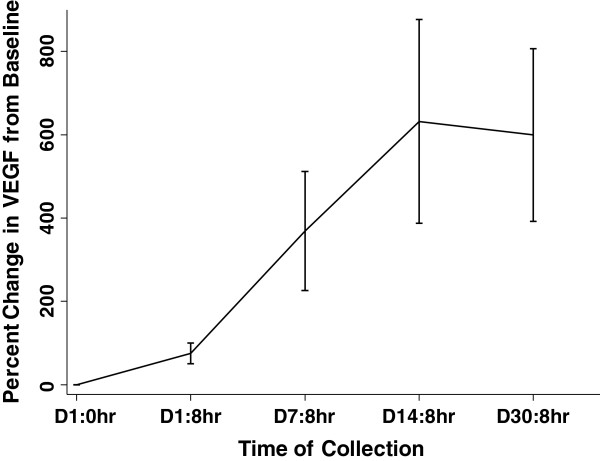
**Change in plasma VEGF in toceranib treated dogs.** Plasma VEGF concentrations were assessed in dogs prior to toceranib therapy then at 8 hours post dosing on days 1, 7, 14, and 30 of the study. The percent change in plasma VEGF over the 30 day study period was then assessed. Plasma VEGF concentrations increased significantly from baseline over the 30 day study period (p < 0.001). Furthermore, plasma VEGF concentrations appeared to plateau over days 14 to 30. Change in VEGF was associated with Cmax (p < 0.001) but not Tmax (p = 0.74).

Plasma was collected from dogs 8 hours post dosing on days 0, 7, 14, and 30 and analyzed by ELISA for VEGF concentrations. As shown in Figure 3, although plasma VEGF baseline concentrations were generally very low in most dogs (10–40 pg/ml) they did increase significantly over the study period (p < 0.001). Furthermore, plasma VEGF concentrations appeared to plateau over days 14 to 30, potentially correlating with the leveling out of toceranib drug peak plasma concentrations. Change in VEGF was associated with Cmax (p < 0.001), but not Tmax (p = 0.74).

## Discussion

Toceranib (Palladia), a multitargeted receptor tyrosine kinase inhibitor was approved by the FDA in 2009 for the treatment of canine MCTs. Based on the pivotal clinical field study the subsequent product label indicated that drug should be administered at 3.25 mg/kg EOD with guidelines for dose reduction as needed to address AEs [1]. However, the initial Phase I clinical trial of toceranib suggested that a dose of 2.5 mg/kg was associated with equivalent biologic activity as that achieved with the MTD [3]. A more recent retrospective study demonstrated significant clinical benefit when toceranib was administered at a median of 2.8 mg/kg [6].

In the current study, dogs with solid tumors were administerd toceranib at an intended dose of 2.5 or 2.75 mg/kg EOD and AEs, response to therapy and plasma concentrations of toceranib and VEGF were assessed. Pharmacokinetic analysis showed that for dogs dosed in the 2.4-2.9 mg/kg range, there was no significant difference among dosing groups with respect to the 6 or 8 hour drug concentrations (presumed Cmax) at each time point tested. Dogs dosed at 2.30-2.39 mg/kg appeared to have lower concentrations of drug at all time points, although there were only 3 dogs in this dose range. Median plasma concentrations of toceranib measured at 6 or 8 hours increased over the first two weeks of therapy for dogs dosed between 2.4-2.9 mg/kg, before leveling off at 100–120 ng/ml. These results differ from those obtained in healthy beagle dogs, where toceranib Cmax was proportional to doses ranging from 2.0 to 6.0 mg/kg [7]. The reason for this is not clear, however it is possible that some differences in drug absorption may occur among dogs with a variety of co-morbidities (such as cancer) compared to that found in laboratory healthy research dogs. Given the studies in mouse models and dogs with MCT demonstrating target inhibition at toceranib plasma concentrations at or above 40 ng/ml [2,4] dosing of toceranib at 2.4-2.9 mg/kg results in plasma concentrations of drug likely to effectively inhibit target receptor tyrosine kinases *in vivo*.

A substantial motivation for assessing whether lower doses of toceranib can be used in dogs with cancer arises from the observed AE profile of the drug. Known AEs include those involving the gastrointestinal tract (anorexia, diarrhea, vomiting), hematologic system (neutropenia, anemia) and neuromuscular system (lameness, weakness), with hepatopathy, proteinuria, hypertension and pancreatitis less frequently reported [1,3]. In the retrospective study of toceranib in solid tumors, while the AEs reported were similar to those established in the pivotal field study, the rates of anorexia, vomiting, lethargy and neutropenia were substantially lower [6].

While the ability to compare toxicities among the published clinical trials evaluating toceranib in dogs with cancer is limited due to variability in dose rates and dosing schedules used, it does appear that the use of lower doses of drug are associated with significantly fewer grade 3 and 4 AEs. Similar to prior studies, the main AEs observed were GI, hematologic (neutropenia) and musculoskeletal in nature and the majority (86%) were either grade 1 or grade 2 in severity (74% and 12%, respectively). However, the number of grade 3 and 4 adverse events was very low, and 3 of these 9 events (pancreatitis, hypertension and ileus) occurred in the dog that experienced an episode initially characterized as pancreatitis that was not confirmed on histopathology following necropsy. Three additional dogs experienced grade 3 and 4 AEs and only the grade 3 lameness was associated with clinical effects. Importantly, no grade 3 or 4 GI adverse events were documented; this is in contrast to the pivotal field study using 3.25 mg/kg dosing in which grade 3 or 4 diarrhea, vomiting and anorexia occurred in 6.9%, 9.2% and 6.2% of treated dogs, respectively [1].

In the current study we documented AEs not previously formally noted with toceranib administration, although these have been reported anecdotally including PLN, hypertension and a presumed hand-foot like syndrome. Hypertension and PLN are AEs known to occur with the administration of anti-angiogenic agents to human cancer patients. In human patients with renal cell carcinoma (RCC) receiving the closely related tyrosine kinase inhibitor sunitinib, 24% developed hypertension and 8% of these were classified as grade 3 in nature [11]. In the current study, only 3 dogs developed hypertension, 2 of which were classified as grade 3. This clinical toxicity may have been underreported as blood pressure was measured only in animals showing signs consistent with systemic hypertension or illness. While the exact mechanism of hypertension associated with toceranib is not clear, it may be directly related to inhibition of VEGF signaling pathways [12]. Additionally, anti-angiogenic drugs used in human patients have been shown to induce increased concentrations of endothelin-1, and this may contribute to the observed hypertension [13-15].

The development of PLN in dogs treated with toceranib has not been described in previous clinical trials, although in these studies routine urinalysis was not performed at study visits. In the current study, urinalysis was similarly only performed at baseline and not repeated unless clinical signs dictated the need to do so. As such, the actual incidence of PLN may have been underreported. In human cancer patients that receive sunitinb the incidence of PLN has been reported to be approximately 2.5% [16-19]. The mechanisms through which VEGF/VEGFR signaling inhibitors cause proteinuria are not well understood, but several have been proposed including the loss of healthy, fenestrated glomerular capillaries which seems to be a direct consequence of blocking VEGFR signaling and possibly disruption of podocyte integrity [16-19].

In this clinical trial, only 8 dogs (20%) had their dose changed throughout the study period (drug holiday and/or dose reduction) compared to 48.3% in the pivotal field study which used the label dose of 3.25 mg/kg [1]. Although limitations exist when comparing data from different studies, it does appear that the reduced AE profile associated with lower doses of toceranib results in fewer changes to the treatment regimen than those that occur using the MTD.

While tumor response was not one of the main objectives of this study, clinical benefit was observed in 36/40 (90%) of the patients, with 12.5% experiencing PR and 77.5% experiencing SD; only 10% of dogs developed PD. Furthermore, 35 of the 40 dogs remained on toceranib for an average of 16.7 weeks after day 30. During this time, one dog with a thyroid carcinoma transitioned from SD to a PR and 3 dogs transitioned to CR (two SCC and one salivary gland adenocarcinoma). These data support the notion that administration of toceranib at doses ranging from 2.4-2.9 mg/kg is associated with meaningful clinical benefit in dogs with cancer.

In human patients administered sunitinib, concentrations of circulating endothelial precursors and plasma VEGF have been shown to increase over time during treatment [20]. Unlike toceranib, sunitinib is generally administered daily for 28 days, followed by a rest period of 14 days, and these concentrations return to baseline during the rest period. It has been hypothesized that continual inhibition of the VEGF/VEGFR signaling axis results in a feedback mechanism promoting an enhanced angiogenic response [21]. The increase in VEGF during treatment with sunitinib or other VEGF/VEGFR inhibitors is generally believed to be a surrogate biomarker of pathway inhibition. In the current study we found that plasma VEGF concentrations increased over time despite using toceranib at the lower dose and giving drug on an EOD basis. This finding further supports the premise that lower doses of toceranib are associated with biologically relevant plasma concentrations of drug.

## Conclusions

In summary, toceranib administration at doses between 2.4-2.9 mg/kg EOD results in plasma drug concentrations associated with expected receptor kinase inhibition. This is supported by the fact that 90% of treated dogs experienced clinical benefit over the 30 day treatment period and 35/40 dogs continued on toceranib following study conclusion. Importantly, AEs associated with toceranib administration at the lower doses were well tolerated with no grade 3 or 4 GI events, compared to those reported with dosing at the MTD of 3.25 mg/kg. Together, these data support the use of toceranib at doses ranging from 2.4-2.9 mg/kg.

## Abbreviations

VEGF: Vascular endothelial growth factor; VEGFR2: Vascular endothelial growth factor receptor-2/KDR/Flk-2; PDGFRα: Platelet derived growth factor receptor-alpha; PDGFRβ: Platelet derived growth factor receptor-beta; KIT: Stem cell factor receptor; RTK: Receptor tyrosine kinase; EOD: Every other day; AGASACA: Apocrine gland anal sac adenocarcinoma; CR: Complete response; PR: Partial response; PD: Progressive disease; SD: Stable disease; Cmax: Maximum plasma concentration of the drug; Tmax: The time after administration of a drug when the maximum plasma concentration is reached; AUC: Area under the curve.

## Competing interests

CL has received honoraria from Pfizer Animal Health (Zoetis) for continuing education events related to toceranib, and for consulting activities related to the use of toceranib in dogs with cancer.

## Authors’ contributions

LFB assisted with patient accrual and management, performed several of the required analyses and wrote the draft of the manuscript. RP, SB, and WC assisted with patient accrual and management. MP performed the statistical analyses for VEGF and PK associations. MY performed the toceranib PK determinations. CAL conceived the clinical trial, oversaw all aspects of the study, and completed the manuscript. All authors read and approved the final manuscript.

## References

[B1] London CA Malpas PB Wood-Follis SL Boucher JF Rusk AW Rosenberg MP Henry CJ Mitchener KL Klein MK Hintermeister JG Multi-center, placebo-controlled, double-blind, randomized study of oral toceranib phosphate (SU11654), a receptor tyrosine kinase inhibitor, for the treatment of dogs with recurrent (either local or distant) mast cell tumor following surgical excision Clin Cancer Res 2009 15 11 3856 3865 10.1158/1078-0432.CCR-08-1860 19470739

[B2] Liao AT Chien MB Shenoy N Mendel DB McMahon G Cherrington JM London CA Inhibition of constitutively active forms of mutant kit by multitargeted indolinone tyrosine kinase inhibitors Blood 2002 100 2 585 593 10.1182/blood-2001-12-0350 12091352

[B3] London CA Hannah AL Zadovoskaya R Chien MB Kollias-Baker C Rosenberg M Downing S Post G Boucher J Shenoy N Phase I dose-escalating study of SU11654, a small molecule receptor tyrosine kinase inhibitor, in dogs with spontaneous malignancies Clin Cancer Res 2003 9 7 2755 2768 12855656

[B4] Mendel DB Laird AD Xin X Louie SG Christensen JG Li G Schreck RE Abrams TJ Ngai TJ Lee LB *In vivo* antitumor activity of SU11248, a novel tyrosine kinase inhibitor targeting vascular endothelial growth factor and platelet-derived growth factor receptors: determination of a pharmacokinetic/pharmacodynamic relationship Clin Cancer Res 2003 9 1 327 337 12538485

[B5] Pryer NK Lee LB Zadovaskaya R Yu X Sukbuntherng J Cherrington JM London CA Proof of target for SU11654: inhibition of KIT phosphorylation in canine mast cell tumors Clin Cancer Res 2003 9 15 5729 5734 14654558

[B6] London C Mathie T Stingle N Clifford C Haney S Klein MK Beaver L Vickery K Vail DM Hershey B Preliminary evidence for biologic activity of toceranib phosphate (Palladia) in solid tumours Vet Comp Oncol 2012 10 3 194 205 10.1111/j.1476-5829.2011.00275.x 22236194 PMC3732378

[B7] Yancey MF Merritt DA Lesman SP Boucher JF Michels GM Pharmacokinetic properties of toceranib phosphate (Palladia, SU11654), a novel tyrosine kinase inhibitor, in laboratory dogs and dogs with mast cell tumors J Vet Pharmacol Ther 2010 33 2 162 171 10.1111/j.1365-2885.2009.01133.x 20444041

[B8] Yancey MF Merritt DA White JA Marsh SA Locuson CW Distribution, metabolism, and excretion of toceranib phosphate (Palladia, SU11654), a novel tyrosine kinase inhibitor, in dogs J Vet Pharmacol Ther 2010 33 2 154 161 10.1111/j.1365-2885.2009.01120.x 20444040

[B9] Veterinary cooperative oncology group - common terminology criteria for adverse events (VCOG-CTCAE) following chemotherapy or biological antineoplastic therapy in dogs and cats v1.1 Vet Comp Oncol 2011 doi:10.1111/j.1476-5829.2011.00283.x [Epub ahead of print] 10.1111/vco.283 28530307

[B10] Eisenhauer EA Therasse P Bogaerts J Schwartz LH Sargent D Ford R Dancey J Arbuck S Gwyther S Mooney M New response evaluation criteria in solid tumours: revised RECIST guideline (version 1.1) Eur J Cancer 2009 45 2 228 247 10.1016/j.ejca.2008.10.026 19097774

[B11] Wood L Sunitinib malate for the treatment of renal cell carcinoma Expert Opin Pharmacother 2012 13 9 1323 1336 10.1517/14656566.2012.689130 22607009

[B12] Zhu X Stergiopoulos K Wu S Risk of hypertension and renal dysfunction with an angiogenesis inhibitor sunitinib: systematic review and meta-analysis Acta Oncol 2009 48 1 9 17 10.1080/02841860802314720 18752081

[B13] Aparicio-Gallego G Afonso-Afonso FJ Leon-Mateos L Firvida-Perez JL Vazquez-Estevez S Lazaro-Quintela M Ramos-Vazquez M Fernandez-Calvo O Campos-Balea B Anton-Aparicio LM Molecular basis of hypertension side effects induced by sunitinib Anticancer Drugs 2011 22 1 1 8 10.1097/CAD.0b013e3283403806 20938340

[B14] Lankhorst S Kappers MH van Esch JH Danser AH van den Meiracker AH Mechanism of hypertension and proteinuria during angiogenesis inhibition: evolving role of endothelin-1 J Hypertens 2013 31 3 444 454 10.1097/HJH.0b013e32835c1d1b 23221987

[B15] Rini BI Cohen DP Lu DR Chen I Hariharan S Gore ME Figlin RA Baum MS Motzer RJ Hypertension as a biomarker of efficacy in patients with metastatic renal cell carcinoma treated with sunitinib J Natl Cancer Inst 2011 103 9 763 773 10.1093/jnci/djr128 21527770 PMC3086879

[B16] Pallotti MC Pantaleo MA Nannini M Centofanti F Fabbrizio B Montanari M Baraldi O Saponara M Lolli C Mandrioli A Development of a nephrotic syndrome in a patient with gastrointestinal stromal tumor during a long-time treatment with sunitinib Case Rep Oncol 2012 5 3 651 656 10.1159/000345946 23275781 PMC3531949

[B17] Turan N Benekli M Ozturk SC Inal S Memis L Guz G Cetin B Buyukberber S Sunitinib- and sorafenib-induced nephrotic syndrome in a patient with gastrointestinal stromal tumor Ann Pharmacother 2012 46 10 e27 10.1345/aph.1R160 23032654

[B18] Kappers MH Smedts FM Horn T van Esch JH Sleijfer S Leijten F Wesseling S Strevens H Jan Danser AH van den Meiracker AH The vascular endothelial growth factor receptor inhibitor sunitinib causes a preeclampsia-like syndrome with activation of the endothelin system Hypertension 2011 58 2 295 302 10.1161/HYPERTENSIONAHA.111.173559 21670421

[B19] Patel TV Morgan JA Demetri GD George S Maki RG Quigley M Humphreys BD A preeclampsia-like syndrome characterized by reversible hypertension and proteinuria induced by the multitargeted kinase inhibitors sunitinib and sorafenib J Natl Cancer Inst 2008 100 4 282 284 10.1093/jnci/djm311 18270341

[B20] Vroling L van der Veldt AA de Haas RR Haanen JB Schuurhuis GJ Kuik DJ van Cruijsen H Verheul HM van den Eertwegh AJ Hoekman K Increased numbers of small circulating endothelial cells in renal cell cancer patients treated with sunitinib Angiogenesis 2009 12 1 69 79 10.1007/s10456-009-9133-9 19212818

[B21] Ebos JM Lee CR Christensen JG Mutsaers AJ Kerbel RS Multiple circulating proangiogenic factors induced by sunitinib malate are tumor-independent and correlate with antitumor efficacy Proc Natl Acad Sci USA 2007 104 43 17069 17074 10.1073/pnas.0708148104 17942672 PMC2040401

